# Time-Domain Investigation of Switchable Filter Wide-Band Antenna for Microwave Breast Imaging

**DOI:** 10.3390/s20154302

**Published:** 2020-08-01

**Authors:** Amir Haider, MuhibUr Rahman, Mahdi Naghshvarianjahromi, Hyung Seok Kim

**Affiliations:** 1 Department of Intelligent Mechatronics Engineering, Sejong University, Seoul 05006, Korea; amirhaider@sejong.ac.kr; 2 Department of Electrical Engineering, Polytechnique Montreal, Montreal, QC H3T 1J4, Canada; muhibur.rahman@polymtl.ca; 3 Department of Electrical and Computer Engineering, McMaster University, Hamilton, ON L8S4L8, Canada; naghshvm@mcmaster.ca

**Keywords:** WiMAX (Worldwide Interoperability for Microwave Access), WLAN (Wireless Local Area Network), IR-UWB, switchable antenna behavior, microwave breast imaging, tumor detection

## Abstract

This paper investigates the time-domain performance of a switchable filter impulse radio ultra-wideband (IR-UWB) antenna for microwave breast imaging applications. A miniaturized CPW-fed integrated filter antenna with switchable performance in the range of the Worldwide Interoperability for Microwave Access (WiMAX) and Wireless Local Area Network (WLAN) bands could operate well within a 3.0 to 11 GHz frequency range. The time-domain performance of the filter antenna was investigated in comparison to that of the designed reference wideband antenna. By comparing both antennas’ time-domain characteristics, it was seen that the switchable filter antenna had good time-domain resolution along with the frequency-domain operation. Additionally, the time-domain investigation revealed that the switchable filter wide-band antenna performed similarly to the reference wide band antenna. This antenna was also utilized for a tumor detection application, and it was seen that the switchable filter wide-band antenna could detect a miniaturized irregularly shaped tumor easily, which is quite promising. Such an antenna with a good time-domain resolution and tumor detection capability will be a good candidate and will find potential applications in microwave breast imaging.

## 1. Introduction

Since 2002, the Federal Communication Commission (FCC) has permitted ultra-wideband (UWB) communication within the frequency range of 3.1–10.6 GHz, which has led to potential applications in the fields of microwave imaging, microwave sensors, surveillance systems, wireless vital sign monitoring, radio-frequency identification (RFID), tumor detection, wireless body area networks, etc. [1,2,3,4,5]. Due to high-speed data rates and good time-domain resolution, such technology has attracted researchers and become one of the hot topics for antenna engineers since the last decade [6]. In microwave imaging sensing applications, wideband radars are explored, and they have found corresponding applications due to multipath effects. Some early work has also been performed on malignant tumor detection and analyzing multilayered breast phantom models [7,8].

In microwave imaging applications, researchers have tried to develop techniques for breast cancer detection, since it is one of the diagnosed cancers in women with the highest chance of incidence. According to a report by the American Cancer Society (ACS), 25% of the cancer diagnoses that occur globally are related to breast cancer. Additionally, the survival rate is almost 80% to 90% at an early stage, which eventually falls to 24% in the corresponding advanced scenarios. Thus, early-stage detection and cost-effective techniques are some of the utmost requirements. Microwave imaging techniques for breast cancer detection have therefore been studied over the past years to meet such requirements [9,10].

Since we know that the UWB spectrum falls within 3–11 GHz, however, we know that certain other standards fall within this spectrum. Such frequency bands create an unavoidable overlap among UWB communication systems and affect their corresponding applications for utilization such as microwave imaging applications. These frequency bands include WLANs operating at 5 to 6 GHz, with sub-standards of lower WLAN having frequencies ranging from 5.15 to 5.35 GHz and upper WLANs ranging from 5.725 to 5.825 GHz, WiMAX that works in the 3.30–3.60 GHz range, X-band for satellite communication functioning in the 7.15–7.60 GHz range, and the International Telecommunication Union (ITU 8-GHz) band functioning at 7.90–8.60 GHz [10,11,12]. Hence, it is of utmost importance to remove this overlap in the frequency domain without distorting the time domain. Frequency domain mitigation has been highly studied in the literature previously, and a lot of different techniques are proposed and investigated in this regard. However, most of them did not investigate its effects in the time domain, which is also an important requirement.

It is well established that the corresponding notched band is generated by introducing slot resonators within the reference antenna such as complementary split-ring resonators, fractal-based different proposed resonators, etc. [13,14,15]. Parasitic resonators, defected ground structures, matching stubs, and filter integration within the reference antenna are also some other techniques that are widely explored [7,16,17,18]. However, all these techniques have limitations in the sense that they perform well within the frequency domain but have a poor resolution in the corresponding time domain. To eliminate such narrowband frequency ranges from within the UWB spectrum, we need a filter antenna that has the characteristics of continuously tuning the corresponding filter band, which will improve the time domain resolution performance. Great interest of researchers has been seen in the frequency domain analysis of band-notched antennas; however, very limited attention has been seen towards time-domain performance. 

Additionally, different antennas have been tried and developed in this regard, to meet the time-domain requirements, but they only have a stationary notched band [14,19,20]. Upon tuning or switching notched bands, their corresponding time-domain performance degrades, and there arises an enormous ringing, which needs to be eliminated by some special post-processing ringing removal technique. The analysis and parameters that should be considered while performing the time-domain analysis of antennas are well described in [21,22]. They analyzed notched band UWB antennas in both the time domain and frequency domain. The band notching behavior is achieved by utilizing a combination of the techniques discussed above. The effect of the modulated Gaussian pulse is studied in different scenarios in detail, and the corresponding time-domain response is provided. A huge amount of ringing has been seen in the time domain as compared to with a UWB antenna without a band notch. One such late time ringing mitigation technique is proposed in [23], which is basically an E-pulse technique that reduces the ringing arising from the notched band’s performance. However, utilizing such a technique for a wide-band antenna and implementing it in microwave imaging made it expensive and complex. 

This paper is focused on the investigation of the time-domain performance of a switchable filter impulse radio ultra-wideband antenna for microwave breast imaging applications. A miniaturized CPW-fed integrated filter antenna with switchable performance in the range of WiMAX and WLAN bands is analyzed, and its corresponding response in the frequency domain is first provided for validation purposes only. The frequency-domain performance shows that the antenna can operate well within a 3.0 to 11 GHz frequency range, having a switchable notched band ranging from 3.3 to 6 GHz upon changing the capacitance of the integrated capacitor within the resonator. The time-domain performance of the filter antenna is investigated in comparison to that of the designed reference wideband antenna. By comparing both antennas, it is seen that the switchable filter antenna has good time-domain resolution along with the frequency domain operation. This antenna is also utilized for tumor detection applications, and it is seen that the switchable filter wide-band antenna can detect a miniaturized irregularly shaped tumor easily, which is quite promising.

The arrangement of this manuscript is as follows: Section 2 briefly presents the selection and design of the reference antenna. Section 3 then briefly presents details regarding the selection and design of a switchable filter antenna. Section 4 deals with the time-domain resolution of the reference and switchable filter antenna with a focus on the measurement setup and S21 calculation. This section is also devoted to important time-domain parameter investigation. Section 5 describes the potential application of the antennas in microwave breast imaging including irregularly shaped miniaturized tumor detection, which is followed by a conclusion section.

## 2. Reference Wide-Band Antenna Design

**Figure 1:**
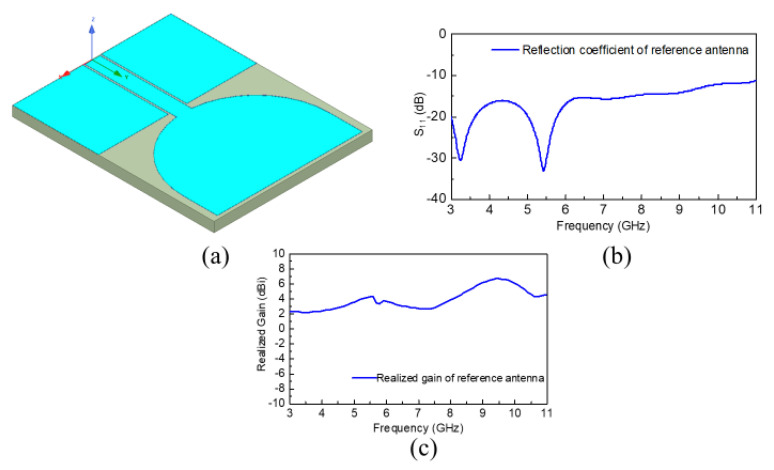
Reference wide-band antenna; (**a**) Geometry of reference wide-band antenna; (**b**) Reflection coefficient of reference wide-band antenna; (**c**) Realized gain of reference wide-band antenna.

The first reference wide-band antenna was developed using a conventional UWB geometry as shown in Figure 1a. As can be observed from Figure 1a, it is a CPW-fed wide-band antenna with the main radiator shaped cylindrically for miniaturization purposes and to achieve good radiation efficiency and realized gain. The substrate used for the reference wide-band antenna design was Rogers RO4003, with a thickness of 1.5 mm and relative dielectric constant, $\varepsilon_{r}=3.38$. The overall antenna dimensions were optimized to $24\times 30.5\;{\mathrm{mm}}^{2}$ including the partial ground plane. This antenna was simulated by means of the commercially available software Ansoft HFSS, and the corresponding reflection coefficient vs. frequency plot is displayed in Figure 1b. Additionally, the corresponding realized gain of the reference wide-band antenna is also provided in Figure 1c, which reveals that this wide-band antenna possesses an acceptable gain within the operating range. The response indicates that this radiator possesses the best operation within the 3 to 11 GHz frequency range.

## 3. Switchable Filter Wide-Band Antenna Design

**Figure 2:**
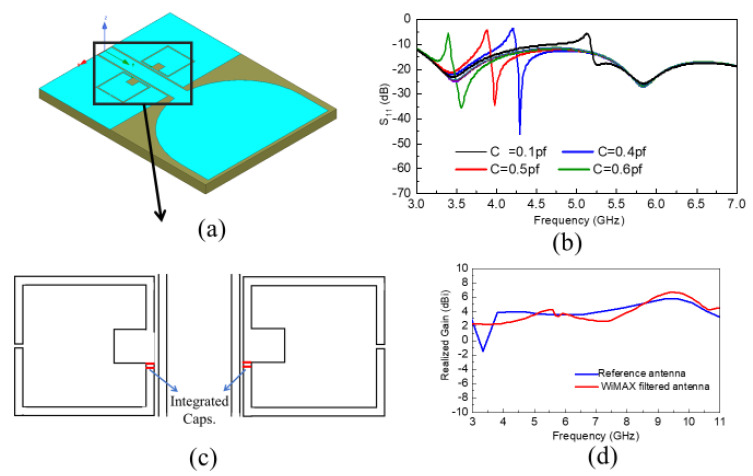
Switchable filter wide-band antenna; (**a**) Geometry of switchable filter wide-band antenna, (**b**) Reflection coefficient of switchable filter wide-band antenna at different capacitor values; (**c**) Zoomed in view of resonators with integrated capacitors; (**d**) Realized gain of reference and Worldwide Interoperability for Microwave Access (WiMAX) filtered wide-band antennas.

**Figure 3:**
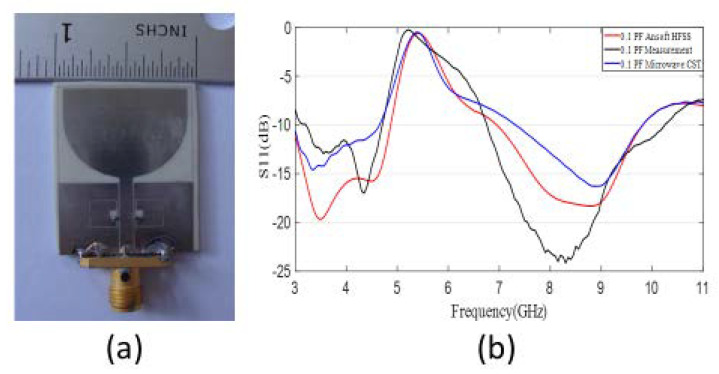
(**a**) Fabricated switchable filter wide-band antenna; (**b**) Reflection coefficient of fabricated switchable filter wide-band antenna with a filter at Wireless Local Area Network (WLAN) range.

Secondly, a switchable filter wide-band antenna was developed and optimized as shown in Figure 2a. As can be seen, a square-shaped filter was integrated within the partial ground plane of the reference antenna to achieve filtered performance. Furthermore, this filtered performance was made switchable by integrating two similar capacitors within the filter, which made the response tunable. This antenna was also fabricated on the Rogers RO4003 substrate with a substrate width and length of $W_{sub}=24\;{\mathrm{mm}}^{2},L_{sub}=30.5\;{\mathrm{mm}}^{2}$, respectively. The utilized switchable filter wide-band antenna was also simulated and measured, and the corresponding reflection coefficient vs. frequency plot is provided in Figure 2b and Figure 3b, respectively. A zoomed-in view of the resonators with integrated capacitors is shown in Figure 2c to demonstrate the position of the capacitors clearly. Additionally, the prototype of the antenna is depicted in Figure 3a. It is observed that the antenna filter performance is switching by altering the capacitance of the corresponding capacitors integrated, without any cost of radiation pattern deterioration and gain due to the placement of the filter in the ground plane. The corresponding realized gain of the reference and WiMAX filtered wide-band antennas is also shown in Figure 2d.

## 4. Time-Domain Investigation

**Figure 4:**
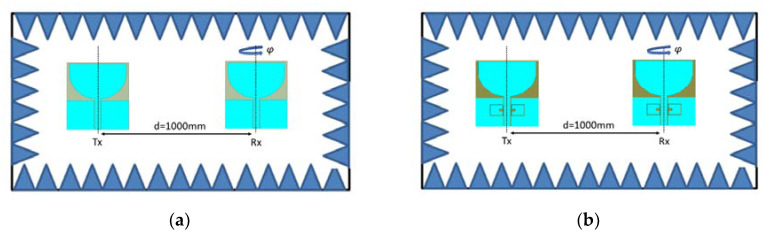
Measurement setup in an anechoic chamber; (**a**) Reference wide-band antenna; (**b**) Switchable filter wide-band antenna.

A time-domain investigation of both the reference and switchable filter antennas was performed in an anechoic chamber as demonstrated in Figure 4a,b, respectively. The measurement setup was comprised of two antennas (either reference or switchable) and was placed in the far-fields of each. As can be observed from Figure 4, the transmitting antenna was placed at *φ* = 0°, while the corresponding receiving antenna was rotated at *φ* = 0°, 90°, 180°, and eventually, the S_21_ response was measured by means of a vector network analyzer (VNA) in each case, which was connected to the Tx and Rx antennas via radio frequency (RF) cables.

Various parameters from the frequency domain are presented as well to characterize the antenna performance in the corresponding time domain. The most important parameter that incorporates the frequency, time, and space together is the fidelity factor (FF), which was analyzed based on [24] and calculated based on our experiment for the reference and switchable filter antenna. The experimental setup is shown in Figure 4a,b for the calculation of the S21 performance of the corresponding reference and switchable filter wide-band antennas, respectively. Then, utilizing Fourier transform, we could calculate the received signal waveform. The fidelity factor could then be calculated using the following equations:

(1) Rx(ω)=[H(ω)Tx(ω)]

where the system transfer function H(ω) is composed of the transfer functions of the transmitting antenna $H_{Tx}$, the receiving antenna $H_{Rx}$, and the channel $H_{Ch}$. Simply, it can be written as $H(\omega )=H_{Tx}H_{Ch}H_{Rx}$.

(2) Rx(t)=IFFT[Rx(ω)]

(3) FF=maxn{∫−∞∞Tx(t)Rx(t+τ)dτ[∫−∞∞|Tx(t)|2dt].[∫−∞∞|Rx(t)|2dt]12}

where Tx(t) represents the transmitted excitation signal, Rx(t) represents the received antenna radiated signal, H(ω) represents the system transfer function, t signifies time, ω signifies frequency, and τ represents the shift in the corresponding transmitted excitation signal and received radiated signal in convolution.

**Figure 5:**
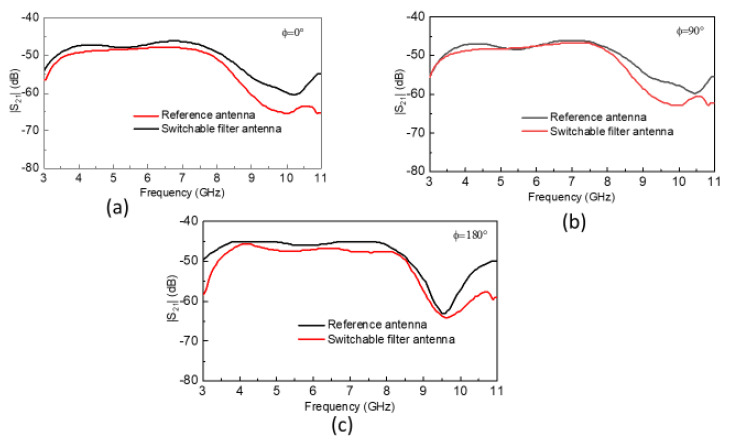
Measurement of S21 characteristics; (**a**) S21 magnitude at *φ* = 0°; (**b**) S21 magnitude at *φ* = 90°; (**c**) S21 magnitude at *φ* = 180°.

The magnitude of the S21 of the reference and switchable filter antennas are shown in Figure 5. The S21 characteristics were almost linear over the whole frequency range for reference antennas at *φ* = 0°, 90°, and 180°. Similarly, the S21 characteristics of the switchable filter antenna were consistent with the reference antenna having a corresponding shift at higher frequencies, which is usual and arises due to differences in the gain of both antennas at higher frequencies and higher-order harmonics. However, the results are valid and acceptable for both antennas, and we can say that both possessed good performance in the time domain as well as in the frequency domain.

**Figure 6:**
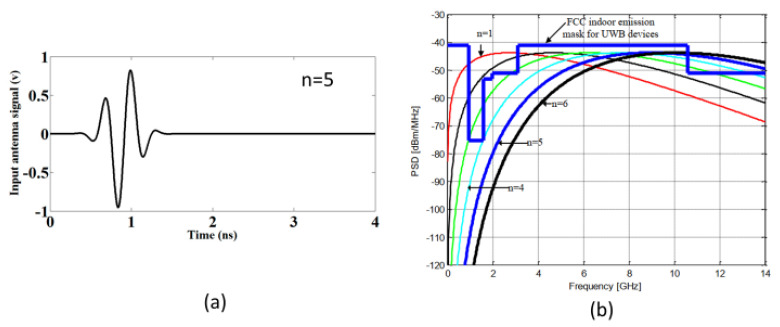
(**a**) Incident pulse used for excitation with n = 5; (**b**) The power spectral density (PSD) of the different derivatives of Gaussian pulses.

**Figure 7:**
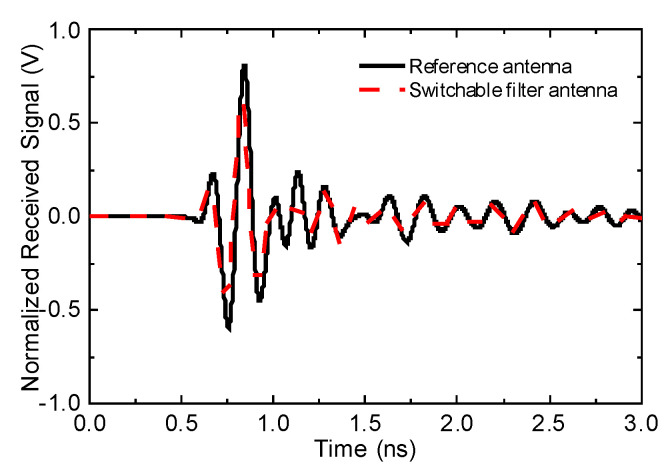
Normalized received signal waveform of reference and switchable antennas.

Figure 6 depicts the incident pulse utilized for excitation, which fell within the FCC indoor emission mask for UWB devices, as clear from Figure 6b, and it is observable from the normalized received waveform in Figure 7 that the switchable and reference antennas are in good agreement, which is quite promising. The corresponding output signals for both antennas were also calculated and are demonstrated in Figure 7. Different order Gaussian pulses are achieved based on the following equation.

(4) $$g_{n}(t)=A_{np}\frac{d^{n}}{dt^{n}}e^{-2\pi }{\left(\frac{t}{\mu_{p}}\right)}^{2}$$

where *A* is the amplitude factor, *n* represents the order, and $\mu_{p}$ is a factor that influences the amplitude and the width of the Gaussian pulse, also called the time normalization factor; the width of a pulse becomes narrower when the $\mu_{p}$ is reduced.

The fidelity factor was calculated from Tx(t) and Rx(t); it is also summarized in Table 1 at different receiving antenna orientations. A fidelity factor greater than 0.8 implies that the corresponding source signal can propagate in a material medium undistorted and, thus, the corresponding received waveform can be completely characterized in such a scenario [25]. The fidelity factor values calculated for the reference and switchable filter antennas are listed in Table 1, both in simulations and measurements. Additionally, the proposed table indicates the fidelity factor at *φ* = 0°, 90°, and 180°, and it is seen that the difference in each orientation is very small and acceptable. Good agreement is observed among the simulation and measurement results here as well. It must be noted here that the reference antenna will always have a good fidelity factor in comparison to the switchable antenna due to the integration of the filter structure within the reference antenna. However, still, the results show that the selected switchable filter antenna has good performance in the time domain and meets all the requirements. 

## 5. Application in Microwave Imaging

**Figure 8:**
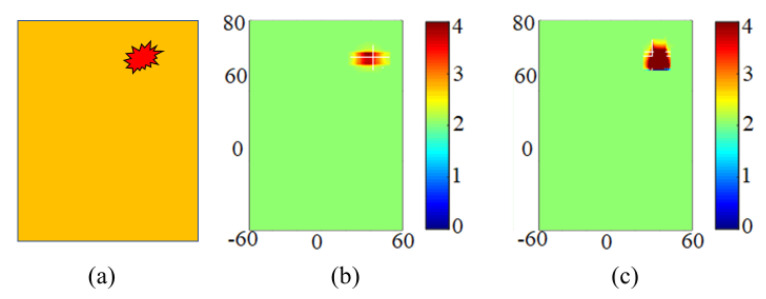
Image of tumor detection by selecting and placing irregular miniaturized tumor; (**a**) Irregularly shaped tumor; (**b**) Reference wide-band antenna; (**c**) Switchable filter wide-band antenna.

The effectiveness of the antenna was tested using a canonical breast model. The electromagnetic properties of the breast tissue and the tumor utilized here were defined as follows. The skin was modeled by using a conductivity σ=4 S/m, a relative permittivity $\varepsilon_{r}=36$, and a thickness of 1.8 mm. Breast tissue was represented by using a first-order Debye model with $\varepsilon_{s}=12$, $\varepsilon_{\infty }=6.5$, and σ=0.14 S/m. The tumor utilized had an irregular, small geometry, as shown in Figure 8a. This is because regular tumors are easy to detect and analyze but irregular tumors have not been studied in detail. Additionally, the practical tumor is not always regular and there are always some variations from the regular geometrical shape. The tumor’s electromagnetic properties are given in terms of a Debye model with $\varepsilon_{s}=51$, $\varepsilon_{\infty }=3.5$, and σ=0.7 S/m. The tumor was assumed to be placed inside the breast model near the skin tissue. Utilizing the microwave imaging technique with the aperture scanning method [26,27,28], the tumor was studied, and it was analyzed for detection. The CST EM solver was used to study the performance of the antenna using the scanning method. The spatial sample rate was selected as 5 mm for the whole phantom model, and the corresponding S21 was obtained. The images that were obtained from the corresponding calibrated S21 at 6 GHz for both the reference and switchable filter antennas are shown in Figure 8b,c, respectively. A tumor was detected as shown in these figures in both cases; however, the exact shape could be viewed and analyzed in much more detail in the switchable case than in the reference one.

The proposed imaging setup comprises two antennas, one for transmitting the UWB signal and another for receiving it to achieve a 2D scan in the (y, z) plane. For the reference antenna case, we used two similar reference antennas for transmitting and receiving, and for the switchable case, we used two similar switchable antennas for transmitting and receiving. The breast was compressed between these similar antennas in each scenario, and the antennas were made movable by scanning the breast completely. The steps involved in image formation were as follows: First, the phantom was placed between two antennas having no tumor, and the S-parameters were recorded in this case at each position and termed as $S_{21}^{back}(y,z)$. Secondly, the phantom was placed between the two antennas having irregularly shaped miniaturized tumors and the S-parameters were again recorded in this case at each position and termed as $S_{21}^{sim}(y,z)$. Finally, the image was extracted by subtracting the S-parameters in the case of no tumor from the corresponding S-parameters in the case of a tumor at each step position and then taking the absolute value using the following equation.

(5) $$\left|S_{21}^{image}(y,z)\right|=\left|S_{21}^{sim}(y,z)-S_{21}^{back}(y,z)\right|$$

## 6. Conclusions

We investigated the time-domain performance of a switchable filter impulse radio ultra-wideband (IR-UWB) antenna for microwave breast imaging applications. A miniaturized CPW-fed integrated filter antenna with switchable performance in the range of the WiMAX and WLAN bands was studied, and its potential applications were explored. The time-domain performance of the filter antenna was investigated in comparison to that of the designed reference wideband antenna, and it was shown that the switchable antenna had a good time-domain resolution. Both antennas were then utilized for a tumor detection application, and it was seen that the switchable filter wide-band antenna could detect a miniaturized irregularly shaped tumor easily, which is quite promising. This antenna will be one of the promising candidates for microwave breast imaging.

## Figures and Tables

**Table 1:** Fidelity factor of the reference and switchable filter antennas.

Fidelity Factor of Both Antennas				
*φ*	Reference Ant. (Sim.)	Reference Ant. (Meas.)	Switchable Filter Ant. (Sim.)	Switchable Filter Ant. (Meas.)
0°	0.93	0.90	0.93	0.89
90°	0.89	0.85	0.86	0.82
180°	0.87	0.85	0.83	0.80
